# Influence of Therapeutic Radiation and Treatment Timing on Dislodgment Resistance of Four Root Canal Sealers: An In Vitro Push‐Out Bond Strength Study

**DOI:** 10.1155/ijod/5302801

**Published:** 2026-10-06

**Authors:** Emshal Malik, Laxmish Mallya, M. S. Athiyamaan, Dilson Lobo, M. Roma, Karthik Shetty, K. Jayaprakash

**Affiliations:** ^1^ Department of Conservative Dentistry and Endodontics Manipal College of Dental Sciences Mangalore Manipal Academy of Higher Education Manipal India manipal.edu; ^2^ Department of Radiation Oncology Kasturba Medical College Mangalore Manipal Academy of Higher Education Manipal India manipal.edu; ^3^ Department of Dental Materials Biomaterials and Research Centre Yenepoya Dental College Yenepoya (Deemed to be University) Mangalore India yenepoya.edu.in

**Keywords:** dislodgment resistance, endodontics failure modes, push-out bond strength, radiation therapy, root canal sealers

## Abstract

**Objectives:**

This in vitro study investigated whether therapeutic radiation and the timing of radiotherapy in relation to endodontic treatment influence the dislodgment resistance of four commonly used root canal sealers bonded to radicular dentin.

**Methods:**

Seventy‐two extracted human single‐rooted teeth were standardized and allocated into four experimental groups according to the sealer used: AH Plus, Cerafill RCS, Sealapex, and Tubliseal. Each group was further divided into three subgroups based on the irradiation protocol: nonirradiated control, root canal treatment followed by radiotherapy, and radiotherapy followed by root canal treatment. Irradiated specimens received a cumulative dose of 70 Gy using a medical linear accelerator. Root canals were instrumented with the ProTaper rotary system and obturated using a single‐cone technique with the assigned sealer. Following specimen preparation, push‐out bond strength (PBS) was measured using a universal testing machine, and the mode of bond failure was evaluated under stereomicroscopy.

**Results:**

AH Plus demonstrated the highest PBS across all experimental conditions, followed by Cerafill RCS, whereas Sealapex and Tubliseal exhibited comparatively lower values. Exposure to therapeutic radiation produced a modest reduction in bond strength; however, neither radiation nor the sequence of radiotherapy and endodontic treatment resulted in statistically significant differences within individual sealer groups. Failure mode analysis showed a predominance of cohesive and mixed failures, with no significant differences among the irradiation protocols.

**Conclusions:**

Within the limitations of this in vitro investigation, therapeutic radiation had only a limited influence on the adhesion of the evaluated root canal sealers to radicular dentin, and the timing of radiotherapy relative to root canal treatment did not significantly affect dislodgment resistance. AH Plus consistently demonstrated superior bonding performance under both irradiated and nonirradiated conditions, indicating its potential suitability for endodontic treatment in patients undergoing head and neck radiotherapy.

## 1. Introduction

Head and neck cancer (HNC) remains one of the most frequently diagnosed malignancies worldwide, with squamous cell carcinoma accounting for the vast majority of cases affecting the oral cavity, pharynx, and larynx. Radiotherapy, administered either alone or in combination with surgery and chemotherapy, is an essential component of treatment because of its effectiveness in improving local disease control and preserving organ function. Despite advances in radiation delivery techniques, exposure of the surrounding normal tissues is unavoidable, leading to several oral and dental complications that may adversely affect the quality of life of cancer survivors [1].

Modern radiotherapy techniques, including intensity‐modulated radiation therapy (IMRT), have substantially reduced radiation‐induced toxicity to adjacent tissues. Nevertheless, teeth and their supporting structures that lie within the radiation field continue to undergo structural and biological alterations. Patients frequently develop acute complications such as oral mucositis, pain, salivary changes, opportunistic infections, and altered taste perception, whereas long‐term sequelae include xerostomia, fibrosis, recurrent infections, sensory disturbances, radiation‐related dental caries, periodontal destruction, and osteoradionecrosis. These complications often complicate restorative and endodontic treatment planning [2, 3].

Radiation‐associated dental caries is characterized by the rapid destruction of cervical, cuspal, and incisal tooth surfaces that are usually resistant to conventional caries. Progressive degradation of the dentino‐enamel junction, accompanied by enamel cracking, mineral loss, collagen degradation, and activation of matrix metalloproteinases, weakens the structural integrity of dental hard tissues. In addition, irradiation promotes the formation of dentinal microcracks, reduces dentin microhardness and elasticity, and alters the organic matrix, thereby increasing susceptibility to bacterial invasion and mechanical failure [2, 4].

Beyond these structural changes, therapeutic radiation may impair pulpal vitality and reduce the mechanical properties of radicular dentin. Irradiated teeth frequently demonstrate reduced microhardness, collagen disorganization, and dentinal microfractures, changes that may increase the likelihood of pulpal necrosis and apical periodontitis, ultimately necessitating root canal treatment. Consequently, comprehensive dental assessment and elimination of existing oral disease before the initiation of radiotherapy are widely recommended to minimize future complications and reduce the need for invasive dental procedures in irradiated patients [3, 5].

Long‐term success of root canal treatment depends on effective cleaning, shaping, and three‐dimensional obturation of the root canal system. An ideal obturation should establish a durable seal that prevents reinfection of the canal space while supporting periapical healing. Because gutta‐percha alone cannot completely adapt to canal irregularities, the performance of the root canal sealer becomes critical in achieving an effective seal [6].

Root canal sealers differ considerably in their chemical composition and clinical behavior. Zinc oxide‐eugenol‐based sealers such as Tubliseal have been extensively used because of their long clinical history, whereas calcium hydroxide‐based materials such as Sealapex were introduced to enhance biological activity and promote hard tissue formation. Epoxy resin‐based AH Plus is regarded as the reference standard because of its excellent adhesion to dentin, dimensional stability, low solubility, and favorable mechanical properties. More recently, calcium silicate‐based bioceramic sealers such as Cerafill RCS have gained popularity owing to their hydrophilic nature, bioactivity, and ability to form hydroxyapatite, thereby creating a chemical bond with radicular dentin [7–9].

The PBS test is widely accepted as a reliable method for evaluating the adhesion of endodontic sealers to the root canal dentin. By measuring the force required to dislodge the obturation material from standardized root sections, this technique provides valuable information regarding the integrity of the sealer–dentin interface and allows comparison of the adhesive performance of different obturation materials under controlled laboratory conditions [10].

Although several investigations have evaluated the influence of therapeutic radiation on dental hard tissues, limited evidence is available regarding the combined effect of radiation exposure and the timing of endodontic treatment on the bonding performance of contemporary root canal sealers. Addressing this knowledge gap may help clinicians select appropriate obturation materials for patients undergoing radiotherapy for head and neck malignancies.

Therefore, the present in vitro study aimed to evaluate the influence of therapeutic radiation and the timing of radiation therapy on the dislodgment resistance of four root canal sealers—AH Plus, Cerafill RCS, Sealapex, and Tubliseal—to radicular dentin. The null hypothesis was that neither therapeutic radiation nor the sequence of radiation relative to root canal treatment would significantly affect the PBS or failure characteristics of the evaluated sealers.

## 2. Materials and Methods

### 2.1. Study Design

This laboratory‐based in vitro experimental study was conducted to investigate the influence of therapeutic radiation and treatment timing on the PBS of four endodontic sealers. Seventy‐two extracted human single‐rooted premolars that satisfied the predefined inclusion and exclusion criteria were included in the investigation.

The specimens were randomly assigned to four experimental groups (*n* = 18) according to the root canal sealer used as follows:

• AH Plus.
• Cerafill RCS.
• Sealapex.
• Tubliseal.

Each sealer group was subsequently divided into three subgroups (*n* = 6) based on the irradiation protocol:

• Group I: nonirradiated control.
• Group II: root canal treatment followed by therapeutic radiation.
• Group III: therapeutic radiation was followed by root canal treatment.

### 2.2. Sample Size Determination

The sample size was estimated using Mead’s resource equation:

E=N−B−T,

where *E* represents the error degrees of freedom, *N* denotes the total number of experimental units, *B* represents the blocking component, and *T* corresponds to the treatment component.

The calculated value indicated that a minimum of five specimens per subgroup would provide an adequate experimental design. To compensate for possible specimen loss during preparation and testing, six samples were included in each subgroup, resulting in a total sample size of 72 specimens.

### 2.3. Specimen Preparation and Endodontic Procedure

Seventy‐two extracted human premolars with a single straight root canal were selected for the study. Teeth presenting with caries, previous endodontic treatment, internal or external resorption, canal calcification, multiple canals, fractures, or developmental anomalies were excluded.

Following selection, the crowns were removed at the cementoenamel junction to standardize the root length to 15 mm. The specimens were disinfected in 5.25% sodium hypochlorite for 30 min and subsequently stored in sterile saline until use.

Each specimen was embedded in self‐curing acrylic resin before instrumentation. The working length was established using a size 15 K‐file, maintaining apical patency throughout instrumentation.

Root canals were prepared using the ProTaper rotary system up to file size F2.

During instrumentation, each canal was irrigated with 5 mL of 5% sodium hypochlorite between successive instruments using a 30‐gauge side‐vented irrigation needle positioned ~1 mm short of the working length.

Passive ultrasonic activation was performed for 30 s between the instrumentation steps. The final irrigation consisted of 17% EDTA for 1 min, followed by a saline rinse.

The canals were dried using sterile paper points and obturated with gutta‐percha and the allocated sealer employing the single‐cone obturation technique. The sealer was introduced into the canal using a lentulospiral. Access openings were sealed temporarily, and all specimens were stored at 37°C to permit the complete setting of the obturation materials.

### 2.4. Irradiation Protocol

Specimens assigned to the irradiation groups were placed in plastic containers containing distilled water with a ~0.5 cm water layer above the samples to facilitate homogeneous radiation exposure.

Radiotherapy was delivered using a medical linear accelerator with a single anterior field measuring 20 × 20 cm^2^ and a source‐to‐surface distance of 80.5 cm.

A cumulative radiation dose of 70 Gy was administered in 35 fractions of 2 Gy per day, 5 days per week, over a 7‐week period using 6‐MV photon beams.

Between irradiation sessions and after completion of radiation exposure, the specimens were stored in artificial saliva (pH 7.0) at 37°C to simulate the oral environment.

### 2.5. PBS Evaluation

After the complete setting of the sealers, each root specimen was embedded in acrylic resin and sectioned perpendicular to its long axis using a diamond disc to obtain slices measuring 1.5 mm in thickness. Slice thickness was verified using a digital micrometer.

PBS testing was performed using a universal testing machine fitted with a 5000 N load cell. A cylindrical plunger measuring 0.8 mm in diameter applied force in an apical‐to‐coronal direction at a crosshead speed of 1 mm/min until displacement of the filling material occurred.

The maximum dislodging force was recorded in Newtons (N), and bond strength was calculated using the following equation:

Push-out bond strength MPa=force N÷bonded surface area mm2.

### 2.6. Failure Mode Analysis

Following bond strength testing, all sections were examined under a stereomicroscope at a 40× magnification.

Failure patterns were classified as follows:

• Adhesive failure: separation at the sealer–dentin interface.
• Cohesive failure: fracture occurring within the sealer material.
• Mixed failure: a combination of adhesive and cohesive failure.

### 2.7. Statistical Analysis

Statistical analysis was performed using the IBM SPSS Statistics software. Continuous variables were expressed as the mean ± standard deviation.

Comparisons among the four sealer groups were carried out using one‐way analysis of variance (ANOVA). When significant differences were detected, Tukey’s post hoc test was used for pairwise comparisons.

Comparisons among irradiation protocols were performed using ANOVA or Welch’s ANOVA, depending on the assumption of homogeneity of variance.

The distribution of failure modes among the experimental groups was evaluated using the chi‐square test.

For all analyses, a *p*‐value < 0.05 was considered statistically significant.

## 3. Results

### 3.1. Comparison of the Bond Strength of Sealers in the Control Group

On comparison of the push‐out bond strength (PBS), the highest mean is seen in AH Plus (26.813333) followed by Cerafill (11.448333), Tubliseal (5.521667), and the least value is seen in Sealapex (3.721667). This parameter shows statistical significance with a test value of 25.653 and a *p* value of <0.001. Intergroup comparison shows that significant differences are seen between Cerafill and AH Plus, AH Plus and Sealapex, and AH Plus and Tubliseal (Tables 1 and 2).

**Table 1:** One‐way analysis of variance (ANOVA) to test the four sealer‐control groups.

Bond strength test	Cerafill (MPa)	AH Plus (MPa)	Sealapex (MPa)	Tubliseal (MPa)	*F*/Welch	*p* value
PBS	11.448 ± 5.225	26.813 ± 6.368	3.722 ± 3.024	5.522 ± 5.097	25.653	<0.001

**Table 2:** Post hoc Tukey test—control.

Bond strength test	Cerafill‐AH Plus (difference [*p* value])	Cerafill‐Sealapex (difference [*p* value])	Cerafill‐Tubliseal (difference [*p* value])	AH Plus‐Sealapex (difference [*p* value])	AH Plus‐Tubliseal (difference [*p* value])	Sealapex‐Tubliseal (difference [*p* value])
PBS	−15.3650000 ^∗^ (<0.001)	7.7266667 (0.069)	5.9266667 (0.213)	23.0916667 ^∗^ (<0.001)	21.2916667 ^∗^ (<0.001)	−1.8 (0.926)

*Note*:  ^∗^ denotes the significance difference between the tested sample.

### 3.2. Comparison of the Bond Strength of Sealers in the RCT Followed by Radiation Groups

On comparison of PBS, the highest mean value was observed for AH Plus (18.805), followed by Ceraseal (11.243333) and Tubliseal (2.96), and the lowest value was observed for Sealapex (2.156667). This parameter shows statistical significance, with a test value of 11.785 ^∗^ and a *p* value of 0.002. Intergroup comparison revealed significant differences between AH Plus and Sealapex and between AH Plus and Tubliseal (Tables 3 and 4).

**Table 3:** One‐way analysis of variance to test the four groups of sealers: RCT‐radiation.

Bond strength test	Cerafill (MPa)	AH Plus (MPa)	Sealapex (MPa)	Tubliseal (MPa)	*F*/Welch	*p* value
PBS	11.243 ± 8.432	18.805 ± 6.82	2.157 ± 1.31	2.96 ± 3.317	11.785 ^∗^	0.002

*Note*:  ^∗^ denotes the significance difference between the tested sample.

**Table 4:** Post hoc Tukey test: RCT‐radiation.

Bond strength test	Cerafill‐AH Plus (difference [*p* value])	Cerafill‐Sealapex (difference [*p* value])	Cerafill‐Tubliseal (difference [*p* value])	AH Plus‐Sealapex (difference [*p* value])	AH plus‐Tubliseal (difference [*p* value])	Sealapex‐Tubliseal (difference [*p* value])
PBS	−7.5616667 (0.133)	9.0866667 (0.054)	8.2833333 (0.088)	16.6483333 ^∗^ (<0.001)	15.8450000 ^∗^ (0.001)	−0.8033333 (0.995)

*Note*:  ^∗^ denotes the significance difference between the tested sample.

### 3.3. Comparison of the Bond Strength of Sealers in Radiation Followed by RCT Group

On comparison of PBS, the highest mean is observed in AH Plus (21.313333), followed by Ceraseal (8.668333), Tubliseal (5.131667), and the least value is observed in Sealapex (3.613333). This parameter shows statistical significance, with a test value of 20.361 and a *p* value of <0.001. Intergroup comparison revealed significant differences between ceraseal and AH Plus, AH Plus and Sealapex, and AH Plus and Tubliseal (Tables 5 and 6).

**Table 5:** One‐way analysis of variance to test the four groups of sealers: radiation‐RCT group.

Bond strength test	Cerafill (MPa)	AH Plus (MPa)	Sealapex (MPa)	Tubliseal (MPa)	*F*/Welch	*p* value
PBS	8.668 ± 1.773	21.313 ± 6.961	3.613 ± 4.036	5.132 ± 2.876	20.361	<0.001

**Table 6:** Post hoc Tukey test: radiation‐RCT.

Bond strength test	Cerafill‐AH Plus (difference [*p* value])	Cerafill‐Sealapex (difference [*p* value])	Cerafill‐Tubliseal (difference [*p* value])	AH Plus‐Sealapex (difference [*p* value])	AH Plus‐Tubliseal (difference [*p* value])	Sealapex‐Tubliseal (difference [*p* value])
PBS	−12.6450000 ^∗^ (<0.001)	5.055 (0.219)	3.5366667 (0.512)	17.7000000 ^∗^ (<0.001)	16.1816667 ^∗^ (<0.001)	−1.5183333 (0.93)

*Note*:  ^∗^ denotes the significance difference between the tested sample.

### 3.4. Comparison of the Three Tested Groups Within Each Sealer

The comparison of PBS among the three experimental groups (control, RCT followed by Radiation, and Radiation followed by RCT) for each sealer was performed using one‐way ANOVA/Welch tests (Table 7).

**Table 7:** Comparison of the three tested groups in each sealer separately.

Push‐out bond strength of sealer	Control (*N* = 6) mean ± SD	RCT + Radiation (*N* = 6) mean ± SD	Radiation + RCT (*N* = 6) mean ± SD	F/Welch statistics	*p* value	Control vs. RCT + Radiation difference (*p* value)	Control vs. Radiation + RCT difference (*p* value)	RCT + Radiation vs. Radiation + RCT difference (*p* value)
Cerafill	11.45 ± 5.22	11.24 ± 8.43	8.67 ± 1.77	0.896^a^	0.448	0.21 (0.998)	2.78 (0.692)	2.58 (0.728)
AH Plus	26.81 ± 6.37	18.81 ± 6.82	21.31 ± 6.96	2.229	0.142	8.01 (0.131)	5.5 (0.357)	−2.51 (0.797)
Sealapex	3.72 ± 3.02	2.16 ± 1.31	3.61 ± 4.04	0.506	0.613	1.57 (0.648)	0.11 (0.998)	−1.46 (0.686)
Tubliseal	5.52 ± 5.1	2.96 ± 3.32	5.13 ± 2.88	0.758	0.486	2.56 (0.504)	0.39 (0.983)	−2.17 (0.607)

^a^Welch test.

For Cerafill, no statistically significant difference was observed among the three groups (*p* = 0.448). Although the control group demonstrated the highest mean PBS (11.45 ± 5.22), followed closely by the RCT + Radiation group (11.24 ± 8.43) and the lowest values in the Radiation + RCT group (8.67 ± 1.77), these differences were not statistically significant. Pairwise comparisons also revealed no significant differences between any of the groups.

For AH Plus, the intergroup comparison similarly showed no statistically significant difference (*p* = 0.142). The control group exhibited the highest mean PBS (26.81 ± 6.37), followed by the Radiation + RCT group (21.31 ± 6.96) and the RCT + Radiation group (18.81 ± 6.82). Despite relatively larger mean differences, particularly between the control and RCT + Radiation groups, none of the pairwise comparisons reached statistical significance.

For Sealapex, there was no statistically significant difference among the three groups (*p* = 0.613). The control group showed slightly higher mean PBS values (3.72 ± 3.02), followed by the Radiation + RCT group (3.61 ± 4.04) and the RCT + Radiation group (2.16 ± 1.31). Pairwise comparisons between all groups were also not statistically significant.

For Tubliseal, the comparison revealed no statistically significant differences among the groups (*p* = 0.486). The highest mean PBS was observed in the control group (5.52 ± 5.10), followed by the Radiation + RCT group (5.13 ± 2.88), while the RCT + Radiation group showed comparatively lower values (2.96 ± 3.32). However, none of these differences were statistically significant on pairwise analysis.

### 3.5. Failure Analysis

Across all sealers, variations in failure modes were observed among the treatment groups, but none were statistically significant. Cerafill showed predominantly cohesive failures (83.3%) in the control and RCT + Radiation groups, with a slight increase in mixed failures (33.3%) in the Radiation + RCT group. AH Plus exhibited mostly cohesive failures (66.7%) in the treated groups, whereas mixed failures were more common in the control group (66.7%). For Sealapex, mixed failures prevailed in the control group (66.7%), whereas cohesive failures increased in the treated groups. Tubliseal showed mixed failures in the control group (66.7%), adhesive failures in the RCT + Radiation group (66.7%), and cohesive failures in the Radiation + RCT group (50%) (Table 8).

**Table 8:** Failure analysis of the push‐out bond strength with different sealers.

Sealer				Failure analysis			Total
				Adhesive	Cohesive	Mixed	
Cerafill	Radiation	Control	Count		5	1	6
			% within radiation		83.3%	16.7%	100.0%
		RCT + Radiation	Count		5	1	6
			% within radiation		83.3%	16.7%	100.0%
		Radiation + RCT	Count		4	2	6
			% within radiation		66.7%	33.3%	100.0%
	Total		Count		14	4	18
			% within radiation		77.8%	22.2%	100.0%

AH Plus	Radiation	Control	Count		2	4	6
			% within radiation		33.3%	66.7%	100.0%
		RCT + Radiation	Count		4	2	6
			% within radiation		66.7%	33.3%	100.0%
		Radiation + RCT	Count		4	2	6
			% within radiation		66.7%	33.3%	100.0%
	Total		Count		10	8	18
			% within Radiation		55.6%	44.4%	100.0%

Sealapex	Radiation	Control	Count	0	2	4	6
			% within radiation	0.0%	33.3%	66.7%	100.0%
		RCT + Radiation	Count	1	4	1	6
			% within radiation	16.7%	66.7%	16.7%	100.0%
		Radiation + RCT	Count	0	5	1	6
			% within radiation	0.0%	83.3%	16.7%	100.0%
	Total		Count	1	11	6	18
			% within radiation	5.6%	61.1%	33.3%	100.0%

Tubliseal	Radiation	Control	Count	1	1	4	6
			% within radiation	16.7%	16.7%	66.7%	100.0%
		RCT + Radiation	Count	4	0	2	6
			% within radiation	66.7%	0.0%	33.3%	100.0%
		Radiation + RCT	Count	1	3	2	6
			% within radiation	16.7%	50.0%	33.3%	100.0%
	Total		Count	6	4	8	18
			% within radiation	33.3%	22.2%	44.4%	100.0%

## 4. Discussion

HNC continues to represent a major global health challenge, with radiotherapy forming an integral component of treatment either as a definitive modality or in combination with surgery and chemotherapy. Approximately three‐quarters of patients receive radiation therapy during the course of their management, most commonly at fractionated doses of 2 Gy per day to cumulative doses ranging between 60 and 70 Gy. Although these regimens improve tumor control, irradiation inevitably affects adjacent normal tissues, including dental hard tissues, creating challenges for restorative and endodontic procedures [11].

Experimental studies have demonstrated that therapeutic radiation can adversely influence the structural integrity of dentin by altering its inorganic and organic components. Radiation‐generated free radicals and reactive oxygen species disrupt the collagen matrix, reduce collagen hydration, and modify the mineralized tissue architecture. Such alterations may compromise the mechanical behavior of dentin and theoretically reduce the adhesion of endodontic sealers to the root canal wall [12–14].

The present investigation evaluated whether these radiation‐induced changes influence the dislodgment resistance of four commonly used root canal sealers. Among the materials tested, AH Plus consistently exhibited the highest PBS under all experimental conditions. This superior performance is most likely related to its epoxy resin chemistry, which promotes intimate adaptation to radicular dentin, low polymerization shrinkage, dimensional stability, and effective mechanical interlocking within dentinal tubules. These characteristics have been widely reported in previous studies and support the continued use of AH Plus as a reference material for evaluating endodontic sealers [15–19].

In contrast, Cerafill RCS demonstrated intermediate bond strength values throughout the study. As a calcium silicate‐based bioceramic sealer, it is capable of interacting chemically with dentin through hydroxyapatite formation and biomineralization, thereby contributing to satisfactory interfacial adaptation. However, its bond strength remained lower than that observed for AH Plus under all irradiation protocols. The findings suggest that although bioactive sealers possess favorable biological characteristics, their immediate resistance to dislodgment may not equal that of epoxy resin‐based sealers under the conditions evaluated in this investigation [17–19].

The comparatively lower bond strengths recorded for Sealapex and Tubliseal may be explained by differences in their composition and physical properties. Calcium hydroxide‐based and zinc oxide‐eugenol‐based sealers generally exhibit greater solubility, reduced cohesive strength, and less effective interaction with radicular dentin than epoxy resin‐based materials. These characteristics may contribute to inferior resistance to displacement during push‐out testing, consistent with the observations reported in previous investigations [15, 16].

An important finding of the present study was that therapeutic radiation produced only a modest reduction in PBS across all experimental groups. Although irradiated specimens generally showed lower mean values than their respective controls, these differences were not statistically significant. Similarly, the sequence in which radiotherapy and root canal treatment were performed did not significantly influence the adhesive performance of any of the evaluated sealers. These findings indicate that, despite measurable structural alterations in irradiated dentin, the bonding ability of the tested sealers remains largely preserved under the experimental conditions employed. Consequently, the null hypothesis could not be rejected.

The evaluation of failure patterns provided additional information regarding the integrity of the sealer–dentin interface. Cohesive and mixed failures predominated across most groups, while adhesive failures were comparatively less frequent. Bioceramic sealers demonstrated a greater proportion of cohesive failures, which may reflect strong interfacial bonding together with fracture occurring within the sealer itself following biomineralization. Conversely, AH Plus exhibited a balanced distribution of cohesive and mixed failures, suggesting that its adhesion to dentin and gutta‐percha remained comparable to its intrinsic cohesive strength. The variable failure patterns observed for Sealapex and Tubliseal are consistent with the inherent physical characteristics of these materials. Importantly, radiation exposure did not significantly alter the overall distribution of failure modes, indicating that irradiation had only a limited effect on the nature of bond failure [20–22].

The findings of this investigation have potential clinical implications for the endodontic management of patients undergoing radiotherapy for head and neck malignancies. Because the timing of radiation relative to root canal treatment did not significantly affect dislodgment resistance, clinicians may not need to modify sealer selection solely on the basis of the treatment sequence. Nevertheless, AH Plus demonstrated consistently superior adhesive performance and therefore appears to remain a reliable option when durable sealing of irradiated root canals is required.

The present study should be interpreted considering certain limitations. As an in vitro investigation, the experimental conditions could not fully reproduce the complex biological environment encountered clinically. Factors such as occlusal loading, intraoral temperature fluctuations, saliva composition, and long‐term aging of restorative materials were not simulated. Furthermore, only the PBS and failure characteristics were evaluated. Additional investigations incorporating aging protocols, microleakage analysis, interfacial ultrastructure, and prospective clinical studies are warranted to further clarify the long‐term behavior of endodontic sealers in irradiated teeth.

Overall, the results indicate that therapeutic radiation has only a limited effect on the adhesion of the evaluated root canal sealers to the radicular dentin. Among the materials investigated, AH Plus consistently demonstrated the greatest resistance to dislodgment, irrespective of irradiation status, supporting its use in endodontic treatment for patients undergoing radiotherapy for HNC.

## 5. Conclusion

Within the limitations of this in vitro study, AH Plus demonstrated significantly greater PBS than Cerafill RCS, Sealapex, and Tubliseal under both irradiated and nonirradiated conditions, indicating superior adhesion to radicular dentin. Cerafill RCS exhibited intermediate bond strength and predominantly cohesive failures, suggesting satisfactory interfacial adaptation despite lower dislodgment resistance than that of AH Plus. In contrast, Sealapex and Tubliseal showed comparatively lower bond strength values with more frequent adhesive and mixed failure patterns.

Therapeutic radiation resulted in a modest reduction in bond strength; however, neither radiation exposure nor the timing of radiotherapy relative to root canal treatment produced statistically significant differences in the adhesive performance or failure characteristics of the evaluated sealers. Based on the present findings, AH Plus appears to provide the most predictable bonding performance and may be considered a reliable obturation material for endodontic treatment in patients receiving radiotherapy for HNC. Nevertheless, well‐designed in vivo and long‐term clinical investigations are required to validate these laboratory findings under clinical conditions.

## Author Contributions

**Emshal Malik**: conceptualization, methodology, investigation, data curation, formal analysis, writing – original draft. **Laxmish Mallya**: conceptualization, methodology, supervision, validation, writing – review and editing, project administration, correspondence. **M. S. Athiyamaan**: methodology, investigation, resources, radiation protocol design, writing – review and editing. **Dilson Lobo**: investigation, resources, validation, writing – review and editing. **M. Roma**: data curation, formal analysis, visualization, writing – review and editing. **Karthik Shetty**: investigation, validation, writing – review and editing. **K. Jayaprakash**: formal analysis, validation, visualization, writing – review and editing.

## Funding

This study did not receive any specific funding.

## Disclosure

All authors have read and approved the final version of the manuscript. Laxmish Mallya, the corresponding author and manuscript guarantor, had full access to all the data in this study and takes complete responsibility for the integrity of the data and the accuracy of the data analysis. The authors affirm that Prevest DenPro Limited had no role in the study design, data collection, data analysis, interpretation of the results, manuscript preparation, or the decision to submit the manuscript for publication. The scientific content and conclusions presented in this manuscript are solely the responsibility of the authors.

## Ethics Statement

Ethical approval for this study was obtained from the Institutional Ethics Committee, Manipal College of Dental Sciences, Mangalore, Manipal Academy of Higher Education, Manipal (Approval Number 23049). Extracted human teeth were collected and used in accordance with institutional ethical guidelines.

## Consent

The authors have nothing to report.

## Conflicts of Interest

The authors declare no conflicts of interest.

## Data Availability

The datasets generated and/or analyzed during the current study are available from the corresponding author upon reasonable request. The data are not publicly available because they contain information that could potentially compromise the privacy and confidentiality of the study. Requests for access to the data may be directed to the corresponding author and will be considered in accordance with applicable ethical and institutional requirements.

## References

[1] Alfouzan A. F. , Radiation Therapy in Head and Neck Cancer, Saudi Medical Journal. (2021) 42, no. 3, 247–254, 10.15537/smj.2021.42.3.20210660. PMC7989258 33632902

[2] Lu H. , Zhao Q. , and Guo J. , et al.Direct Radiation-Induced Effects on Dental Hard Tissue, Radiation Oncology. (2019) 14, no. 1, 10.1186/s13014-019-1208-1. PMC6329176 30635005

[3] Sroussi H. Y. , Epstein J. B. , and Bensadoun R. J. , et al.Common Oral Complications of Head and Neck Cancer Radiation Therapy: Mucositis, Infections, Saliva Change, Fibrosis, Sensory Dysfunctions, Dental Caries, Periodontal Disease, and Osteoradionecrosis, Cancer Medicine. (2017) 6, no. 12, 2918–2931, 10.1002/cam4.1221. PMC5727249 29071801

[4] Walker M. P. , Wichman B. , Cheng A. L. , Coster J. , and Williams K. B. , Impact of Radiotherapy Dose on Dentition Breakdown in Head and Neck Cancer Patients, Practical Radiation Oncology. (2011) 1, no. 3, 142–148, 10.1016/j.prro.2011.03.003. PMC3156461 21857887

[5] Gulderen O. , Saricam E. , Gökhan Açikgöz S. , and Tezcan Y. , Effects of Radiotherapy Dose and Application Time on the Load-to-Failure Values of Teeth Filled With Different Sealers, BMC Oral Health. (2024) 24, no. 1, 10.1186/s12903-024-05029-4, 1260. PMC11495028 39434091

[6] Gusiyska A. and Dyulgerova E. , Clinical Approaches to the Three-Dimensional Endodontic Obturation Protocol for Teeth With Periapical Bone Lesions, 2023, Multidisciplinary Digital Publishing Institute (MDPI).

[7] Komabayashi T. , Colmenar D. , Cvach N. , Bhat A. , Primus C. , and Imai Y. , Comprehensive Review of Current Endodontic Sealers, Dental Materials Journal. (2020) 39, no. 5, 703–720, 10.4012/dmj.2019-288. 32213767

[8] Yaduka P. , Kataki R. , Roy D. , Das L. , and Goswami S. , Effects of Radiation Therapy on the Dislocation Resistance of Root Canal Sealers Applied to Dentin and the Sealer-Dentin Interface: A Pilot Study, Restorative Dentistry & Endodontics. (2021) 46, no. 2, 10.5395/rde.2021.46.e22, e22. PMC8170382 34123758

[9] Agrawal T. S. , Singh S. , Podar R. S. , Kulkarni G. , Gadkari A. , and Agarwal N. , In Vitro Comparative Evaluation of Apical Leakage Using a Bioceramic Sealer With Three Different Obturating Techniques: A Glucose Leakage Model, Journal of Conservative Dentistry and Endodontics, Jan. (2024) 27, no. 1, 76–81, 10.4103/jcd.jcd_303_23. PMC10880467 38389733

[10] Moinuddin M. , Prasad L. , Ramachandruni N. , Kamishetty S. , and Cherkupalli R. , Comparison of Push-Out Bond Strength of Three Different Obturating Systems to Intraradicular Dentin: An in Vitro Study, Contemporary Clinical Dentistry. (2019) 10, no. 4, 631–636. 10.4103/ccd.ccd_640_18 PMC7390420 32792822

[11] Bodrumlu E. , Avsar A. , Meydan A. D. , and Tuloglu M. , Can Radiotherapy Affect the Apical Sealing Ability of Resin-Based Root Canal Sealers?, The Journal of the American Dental Association. (2009) 140, no. 3, 326–330, 10.14219/jada.archive.2009.0162. 19255177

[12] Gonçalves L. M. N. , Palma-Dibb R. G. , and Paula-Silva F. W. G. , et al.Radiation Therapy Alters Microhardness and Microstructure of Enamel and Dentin of Permanent Human Teeth, Journal of Dentistry. (2014) 42, no. 8, 986–992, 10.1016/j.jdent.2014.05.011. 24887361

[13] Martins C. V. , Leoni G. B. , and Oliveira H. F. , et al.Influence of Therapeutic Cancer Radiation on the Bond Strength of an Epoxy- or an MTA-Based Sealer to Root Dentine, International Endodontic Journal. (2016) 49, no. 11, 1065–1072. 10.1111/iej.12556 26426161

[14] Perdigão J. , Van Meerbeek B. , Lopes M. M. , and Ambrose W. W. , The Effect of a Re-Wetting Agent on Dentin Bonding [Internet], https://www.elsevier.com/locate/dental. 10.1016/s0109-5641(99)00049-4 10551097

[15] Fouda S. , Labib A. , Shaheen N. , Abd-Elbary N. , and Alhadainy H. , Effect of Radiotherapy on Bond Strength of Different Endodontic Sealers to Root Dentin, Tanta Dental Journal. (2020) 17, no. 3, 10.4103/tdj.tdj_48_19.

[16] Lee K. W. , Williams M. C. , Camps J. J. , and Pashley D. H. , Adhesion of Endodontic Sealers to Dentin and Gutta-Percha, 2002. 10.1097/00004770-200210000-00002 12398164

[17] Abdollahi A. A. , Seyyedi S. A. , Khodaie A. , and Afshar N. N. , Push-Out Bond Strength of a New Bioceramic-Based Root Canal Sealer, Journal of Dental Materials and Techniques. (2024) 13, no. 1, 26–32.

[18] Gade V. J. , Belsare L. D. , Patil S. , Bhede R. , and Gade J. R. , Evaluation of Push-Out Bond Strength of Endosequence BC Sealer With Lateral Condensation and Thermoplasticized Technique: An in Vitro Study, Journal of Conservative Dentistry. (2015) 18, no. 2, 124–127. 10.4103/0972-0707.153075 PMC4379651 25829690

[19] Carvalho N. K. , Prado M. C. , and Senna P. M. , et al.Do Smear-Layer Removal Agents Affect the Push-Out Bond Strength of Calcium Silicate-Based Endodontic Sealers?, International Endodontic Journal. (2017) 50, no. 6, 612–619, 10.1111/iej.12662. 27214521

[20] Yap W. Y. , Che Ab Aziz Z. A. , Azami N. H. , Al-Haddad A. Y. , and Khan A. A. , An in Vitro Comparison of Bond Strength of Different Sealers/Obturation Systems to Root Dentin Using the Push-Out Test at 2 Weeks and 3 Months After Obturation, Medical Principles and Practice. (2017) 26, no. 5, 464–469. 10.1159/000481623 PMC5757529 28934753

[21] Frasquetti K. S. , Piasecki L. , Kowalczuck A. , Carneiro E. , Westphalen V. P. D. , and Neto U. X. D. S. , Effect of Different Root Canal Drying Protocols on the Bond Strength of Two Bioceramic Sealers, European Journal of Dentistry. (2023) 17, no. 4, 1229–1234. 10.1055/s-0042-1758807 PMC10756819 36535659

[22] Retana-Lobo C. , Tanomaru-Filho M. , Guerreiro-Tanomaru J. M. , Benavides-García M. , Hernández-Meza E. , and Reyes-Carmona J. , Push-Out Bond Strength, Characterization, and Ion Release of Premixed and Powder-Liquid Bioceramic Sealers With or Without Gutta-Percha, Scanning. (2021) 2021, 10.1155/2021/6617930, 6617930. PMC8121597 34040690

